# Supplementary material for: Cerebral-Organoid-Derived Exosomes Alleviate Oxidative Stress and Promote LMX1A-Dependent Dopaminergic Differentiation
Source: Int J Mol Sci. 2023 Jul 4;24(13):11048. doi: 10.3390/ijms241311048 (PMC10341736; doi:10.3390/ijms241311048)
Supplement: Supplementary file 1 [file ijms-24-11048-s001.zip › ijms-2455890-supplementary.pdf]

Supplementary Materials

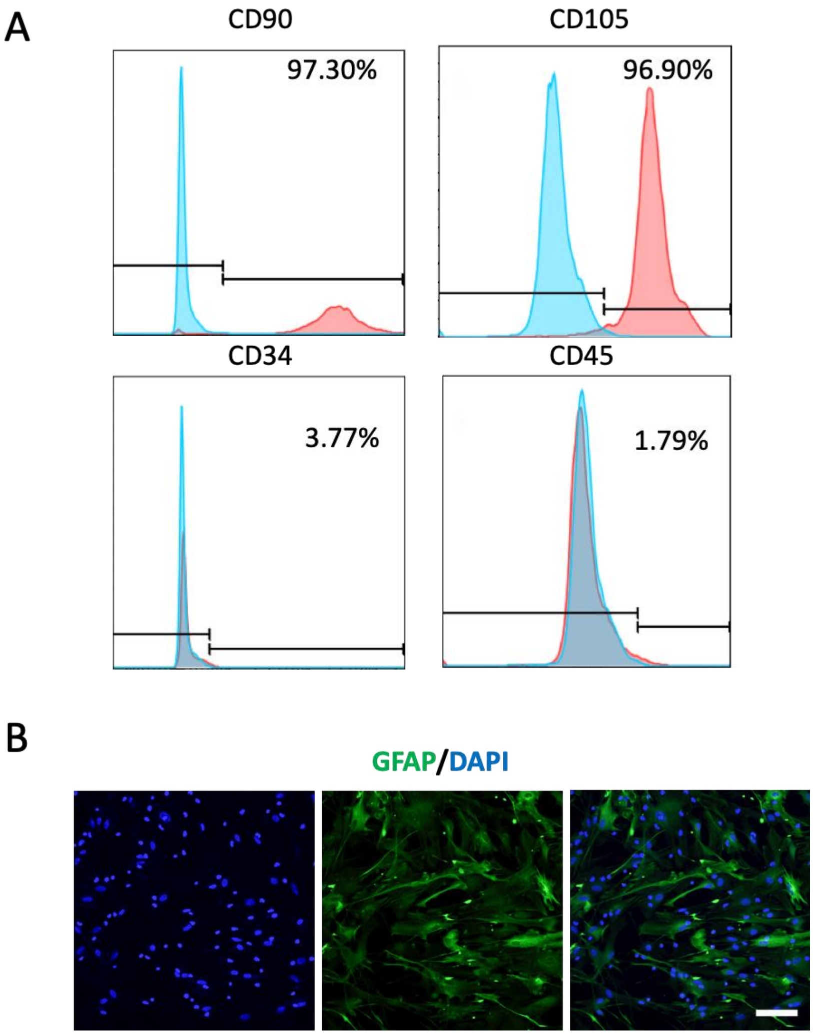

Figure S1. Characterization of primary cultured MSCs and rat midbrain AS. (A) MSCs were stained with surface markers CD90, CD105, CD34, and CD45 and subjected to flow cytometry analysis. ~97% of cells are immunoreactive to mesenchymal stem cell markers CD90 and CD105 and negative for hematopoietic markers CD34 and CD45. (B) Immunostaining analysis showed that nearly 95% of cells in the primary astrocytic culture were immunopositive for astrocytic marker GFAP. Scale bars: 100 μm.

Table S1. The specific primer sequences (5′–3′)

Genes	Forward primer	Reverse primer
Human		
gpadh	GGAGCGAGATCCCTCCAAAAT	GGCTGTTGTCAT-ACTTCTCATGG
nt-4	CTGTGTGCGATGCAGTCAGT	TGCAGCGGGTTTCAAAGAAGT
bdnf	GGCTTGACATCATTGGCTGAC	CATTGGGCCGAACTTTCTGGT
gdnf	GGCAGTGCTTCCTAGAAGAGA	AAGACACAACCCCGGTTTTTG
lmx1a	GCAAAGGGGACTATGAGAAGGA	CGTTTGGGGCGCTTATGGT
wnt1	CGATGGTGGGGTATTGTGAAC	CCGGATTTTGGCGTATCAGAC
en1	TTCCAGGCAAACCGCTACATC	ACTCGCTCTCGTCTTTGTCCT
corin	CCAAAGCCGGTCTTGAGAG	GAGGAGGTTAGCAGTCGCC
mash1	CCAAGCAAGTCAAGCGACAG	TAGCCAAAGCCGCTGAAGTT
ptbp1	CTCACCAGCCTCAACGTCAA	TTATACCAGGTGCACCGAAGG

pitx3	CAGAGGACGGTTCGCTGAAAA	AGCTGCCTTTGCATAGCTCG
nurr1	ACCACTCTTCGGGAGAATACA	GGCATTTGGTACAAGCAAGGT
Rat		
β-actin	ATGGATGACGATATCGCTGCG	GTAGGAGTCCTTCTGACCCAT
apaf-1	ACTTGTCGGCCCTGCGCATC	GGGGCGAACGACTAAGCGGG
bax	TTGCTACAGGGTTTCATCC	GTCCAGTTCATCGCCAAT
bcl-2	GTCATAACTAAAGACACCCC	TTCATCTCCAGTATCCCACTC
caspase3	CGGAGCTTGGAACGCGA	ACACAAGCCCATTTCAGGGTA
p53	GTCGGCTCCGACTATACCACTATC	CTCTCTTTGCACTCCCTGGGGG
